# Workforce agility: a systematic literature review and research agenda

**DOI:** 10.3389/fpsyg.2024.1376399

**Published:** 2024-09-11

**Authors:** Devi Alviani, Hilmiana, Sunu Widianto, Wa Ode Zusnita Muizu

**Affiliations:** Department of Management, Faculty of Economics and Business, Universitas Padjadjaran, Bandung, Indonesia

**Keywords:** agile workforce, workforce agility, adaptivity, proactive, resilience

## Abstract

**Introduction:**

The definition and operationalization of workforce agility, initially associated with multitasking abilities, have evolved to encompass aspects of adaptability, proactivity, and resilience, which are now widely accepted. However, some authors have expanded this concept by adding elements such as intelligence, collaboration, and social support, leading to confusion and disagreement on how to consistently measure workforce agility. Furthermore, the literature emphasizes the importance of workforce agility in achieving business goals and adopting innovative management models, yet it provides limited structured guidance for future research.

**Methods:**

This study uses Scopus and Web of Science as the primary databases. The search was not limited to a specific period but included articles up to 2024, with an initial sample of 176 articles. After a screening process based on inclusion and exclusion criteria, 74 articles were included in the thematic analysis and data synthesis.

**Results:**

Workforce agility has been positioned as an independent, mediating, moderating, and dependent variable in various studies. Most studies examine workforce agility at the individual level, with only about 10 studies exploring this aspect at the organizational level. However, no research has specifically explored workforce agility at the team level to date. The primary objective of workforce agility research is to expand and connect theories with diverse methodological approaches, including quantitative, qualitative, and mixed methods. As a result, theoretical foundations and inter-variable relationships are established to identify recommendations for future research.

**Discussion:**

More than 25 fundamental theories have been identified and categorized into nine groups. These nine groups were then reformulated into four general theories: Organizational and Management Theory, Communication and Social Interaction Theory, Behavioral and Learning Theory, and Economic Theory. Based on previous research, several recommendations for future research have been outlined, including conducting longitudinal studies, integrating mixed methods, considering the global cultural context, expanding research samples, developing conceptual models, exploring mediating and moderating variables, developing workforce agility theories, creating efficient evaluation methods, and implementing multilevel models.

## 1 Introduction

Industry 5.0 is recognized as the digital era that creates a highly dynamic business environment, compelling organizations to continually enhance strategies, processes, and management practices to survive and achieve a competitive advantage (Ito et al., 2023). This situation opposes the traditional management approach, which often has limitations in problem-solving. Such an approach tends to be hierarchical and structured, making it less responsive to innovation, socio-cultural changes, and management practice updates. To address these challenges, a transformation in managerial processes is needed to become more creative and flexible (Da Costa et al., 2022). The primary focus of this transformation lies in developing workforce competencies, which need to be trained to demonstrate agility (Sherehiy and Karwowski, 2014; Doeze Jager-van Vliet et al., 2019).

Workforce agility is the ability to efficiently respond or continuously adapt to changes (Al Hammouri et al., 2023). Several studies, such as Srivastava and Gupta (2022), Muduli (2016), and Pitafi et al. (2019), have linked workforce agility with workplace spirituality, training and development, communication quality, psychological empowerment, networking ties, and various other factors (Muduli, 2016; Pitafi et al., 2019; Srivastava and Gupta, 2022). Although workforce agility has garnered attention in both academic and practical domains, research on this topic is in the early stage of development, and the concept is relatively new (Petermann and Zacher, 2022). Therefore, there is insufficient investigation into organizational characteristics and human resource management that drive workforce agility (Sherehiy and Karwowski, 2014; Harsch and Festing, 2020). Several reasons may explain this situation. First, there is a lack of consensus on the definition and operationalization of workforce agility. As a construct of the capability to face change, the concept of workforce agility originally referred to the proportion of operators capable of performing multiple tasks (Sumukadas and Sawhney, 2004) and later gained popularity with the domains of adaptivity, proactivity, and resilience (Alavi et al., 2014; Sherehiy and Karwowski, 2014). Although most authors agree with the concept (Zandi et al., 2022; Zhang et al., 2022; Heidt et al., 2023), some authors have expanded it to include individual renewal, collaboration, creating positive relationships, openness to experience and social support (Braun et al., 2017), intelligence, competence, collaboration, resilience, and culture (Herlina et al., 2021). As a result, a comprehensive understanding of workforce agility has not been fully achieved. It seems biased, likely due to these features’ resemblance to other more popular constructs such as work proactive behavior (Morrison and Phelps, 1999), proactive personality (Bateman and Crant, 1993), and openness to experience as one of the facets of the big five personality traits (McCrae and Costa, 1997). This confusion has further led to disagreements on how to measure workforce agility. Second, agility needs further development to deepen understanding of the existing theoretical frameworks, in line with Post et al. (2020) advocating for more remarkable theoretical progress (Salmen and Festing, 2022). Third, there is a need to create a conceptual model to clarify understanding, theory development, and hypothesis testing by highlighting sixteen drivers of workforce agility and expanding it to other disciplines (Menon and Suresh, 2022). Fourth, there are gaps related to the role of managers in developing agility and the importance of aligning investment with expected outcomes (Tessarini Junior and Saltorato, 2021). Fifth, an understanding of workforce agility involving factors such as workplace spirituality, adaptive performance, proactive performance, role flexibility, learning agility, and resilience needs to be expanded (Paul et al., 2020; Park and Park, 2021). As a result, some fundamental questions regarding theorization remain unanswered. For instance, there is a lack of precise and in-depth understanding of the antecedents, mediator mechanisms, moderator contingencies, and theories underlying these relationships. This understanding is essential to consider the role of workforce agility in determining performance (Baraei and Mirzaei, 2018; Munteanu et al., 2020; Varshney and Varshney, 2020). Workforce agility facilitates the rapid achievement of business objectives by adopting innovative management models and organizational cultures that suggest value addition, continuous improvement, and collaborative problem-solving (Alahyari et al., 2019). Moreover, the literature has not presented recommended guidelines regarding workforce agility and potential research gaps as directions for future research in a structured manner. These limitations can hinder filling existing knowledge gaps and developing a more comprehensive understanding of workforce agility.

This research aimed to (1) examine existing literature and integrate available insights to present a comprehensive overview of the research models researched to date and the underlying theoretical understanding; (2) evaluate the suggestions of research in the context of workforce agility to date, showing new topics and identifying remaining knowledge gaps. More specifically, in this systematic literature review, we address two questions:

RQ1: What are the antecedents, mechanisms (mediators), outcomes, and contingencies (moderators) of workforce agility, and which underpinning theories explain the suggested relationships?

RQ2: What have previous researchers suggested regarding workforce agility literature, and what potential avenues are available for further development and expansion?

These answers to the questions are expected to contribute significantly to literature in several aspects. First, this study is a response to the call for more profound research in considering the issue of workforce agility (Sherehiy and Karwowski, 2014; Harsch and Festing, 2020; Petermann and Zacher, 2022). Second, the current research reaffirms a deeper understanding of the mediating mechanisms, moderating, and influencing factors of workforce agility, as well as the supporting theoretical framework (Baraei and Mirzaei, 2018; Munteanu et al., 2020; Varshney and Varshney, 2020; Heidt et al., 2023; Rasheed et al., 2023; Talwar et al., 2023). Third, concrete ideas for further investigation were offered, expected to fill the identified knowledge gaps. An SLR methodology was applied to evaluate 74 peer-reviewed and indexed articles in the Scopus and Web of Science database over two decades. This study is structured as follows. The first section is the introduction, and the second is the method section, which consists of four key steps in our review. The third section presents the results, discussing a descriptive analysis of the articles under research. The fourth section presents a discussion of the reviewed literature. Finally, the last section identifies research limitations, guiding readers toward future research directions and conclusions.

## 2 Method

Tranfield et al. (2003) and Leonidou et al. (2020) provided guidelines for conducting a systematic review consisting of four main steps: (1) defining research questions, (2) defining the review protocol, (3) conducting descriptive analysis of the results, and (4) performing thematic analysis and synthesizing the collected data. The following subsections explain these four steps.

### 2.1 Defining research questions

This research aims to systematically synthesize and integrate existing research on workforce agility by addressing two primary research questions. The first question identifies the antecedents, mechanisms (mediators), outcomes, and contingencies (moderators) of workforce agility and the theoretical frameworks underlying these relationships. The second question explores previous findings in the workforce agility literature and identifies potential avenues for further development and expansion.

### 2.2 Defining the review protocol

This study applies several inclusion and exclusion criteria. Scopus and Web of Science were selected as the primary databases to narrow the scope of the research (Hung and Law, 2011; Zott et al., 2011; Battisti et al., 2019; Castañer and Oliveira, 2020). These were chosen due to their broad coverage and relatively well-maintained quality control (Harzing and Alakangas, 2016; Schotten et al., 2017). In the subsequent step, titles, abstracts, and keywords of articles were explored using keyword combinations based on “Agile Employee,” “Employee Agility,” “Agile Worker,” “Agile Workforce,” and “Workforce Agility.” To include all pertinent research without temporal constraints, the search was not confined to a specific period but included articles up to 2024. The next step is to prioritize contributions with the highest impact and visibility. “Articles” are specifically chosen as the type of document, and “academic journals” (peer-reviewed) as the type of publication (Battisti et al., 2021; Christofi et al., 2021). Scientific publication articles are prioritized because they are considered the most reliable and authoritative sources of information (Anderson et al., 2020; Smela et al., 2023). Articles have been peer-reviewed and undergone a rigorous evaluation, so the research was conducted with high scientific rigor. Also, their findings are accurate and trustworthy (Anderson et al., 2020; Azarian et al., 2023; Smela et al., 2023). Consequently, editorials, perspectives, conference proceedings, book chapters, or other contributions lacking peer review scrutiny were excluded (López-Duarte et al., 2016). Inclusion criteria were limited to contributions in the English language, excluding those in other languages (Follmer and Jones, 2018), both due to the language constraints of research experts (Battisti et al., 2021) and the perceived limited impact of such contributions on international academic discourse (Gallardo-Gallardo and Thunnissen, 2016). In addition, only articles with available full text were considered (Vrontis and Christofi, 2021). The initial putative sample of articles meeting these criteria amounted to 176.

Various exclusion criteria were implemented in the review process. Firstly, in accordance with the typical procedure for tracking research trends in an SLR study (Radaelli and Sitton-Kent, 2016; Battisti et al., 2021), duplicate articles only partially written in English (e.g., abstract in English, text in another language) were eliminated (Sousa et al., 2008). Secondly, titles, abstracts, and keywords of articles were scrutinized, excluding research that did not correlate with the primary objective of the review (Christofi et al., 2017). At this stage, a flexible and inclusive method was adopted. We conducted a detailed assessment to determine whether the research partially or entirely focuses on the research questions. Research focusing on the research questions would proceed to the next stage (Vrontis and Christofi, 2021). Consequently, the number of articles meeting the exclusion criteria in the sample was reduced to 97.

### 2.3 Conducting descriptive analysis of the results

Based on a thorough reading of the full texts, the contributions crucial for comprehending the topic of discourse were identified (Leonidou et al., 2020). Several studies that did not specifically address workforce agility were excluded. Following the method outlined by Aguinis et al. (2018), a calibration process was adopted to select the documents. Only articles adding depth to understanding workforce agility and its causation were included. Articles without a primary focus on workforce agility but still provided insights into the analyzed research area were incorporated. Due to these considerations, 23 articles were excluded, resulting in a sample of 74.

### 2.4 Thematic analysis and synthesizing data

Data extraction in an SLR requires transparency and a systematic method (Kraus et al., 2020). Manual synthesis was performed (Rose et al., 2011) using Microsoft Excel software (Danese et al., 2018), as detailed in the third section of this research, where the data were organized. Thematic analysis was subsequently conducted to synthesize the results into an integrative framework (Bertello et al., 2022).

Thematic analysis was conducted by recognizing, reading, and comprehensively understanding the data. A total of 74 articles were reviewed, focusing on gathering information related to how theories shaped the framework, the systematic method used to achieve the research objectives. Then, the data were coded by labeling relevant segments. Following other systematic literature reviews, we conducted open, axial, and selective coding (Wolfswinkel et al., 2013; A’yuninnisa et al., 2023). After that, we searched for common themes from those codes, reviewed the themes to ensure relevance and interrelation, and named the themes descriptively. In this context, we looked for the most commonly used theories to connect variables and form a nomological network. We also analyzed recommendations for future research and organizations. The recommendations were tabulated and presented as key points, explained using general terms rather than the actual terminology from the reviewed articles. Finally, a report was written to describe these themes in order to answer the research questions.

The analysis focused on two aspects of the articles: attributes and findings. Descriptive statistics were adopted to analyze attributes such as variables positions, units of analysis, research objectives, and previous research methodologies. The theoretical framework and its relationship and future research roadmap were also identified. Visual shows in the form of tables were provided to aid in understanding the publication attributes included in this review.

**Figure 1:**
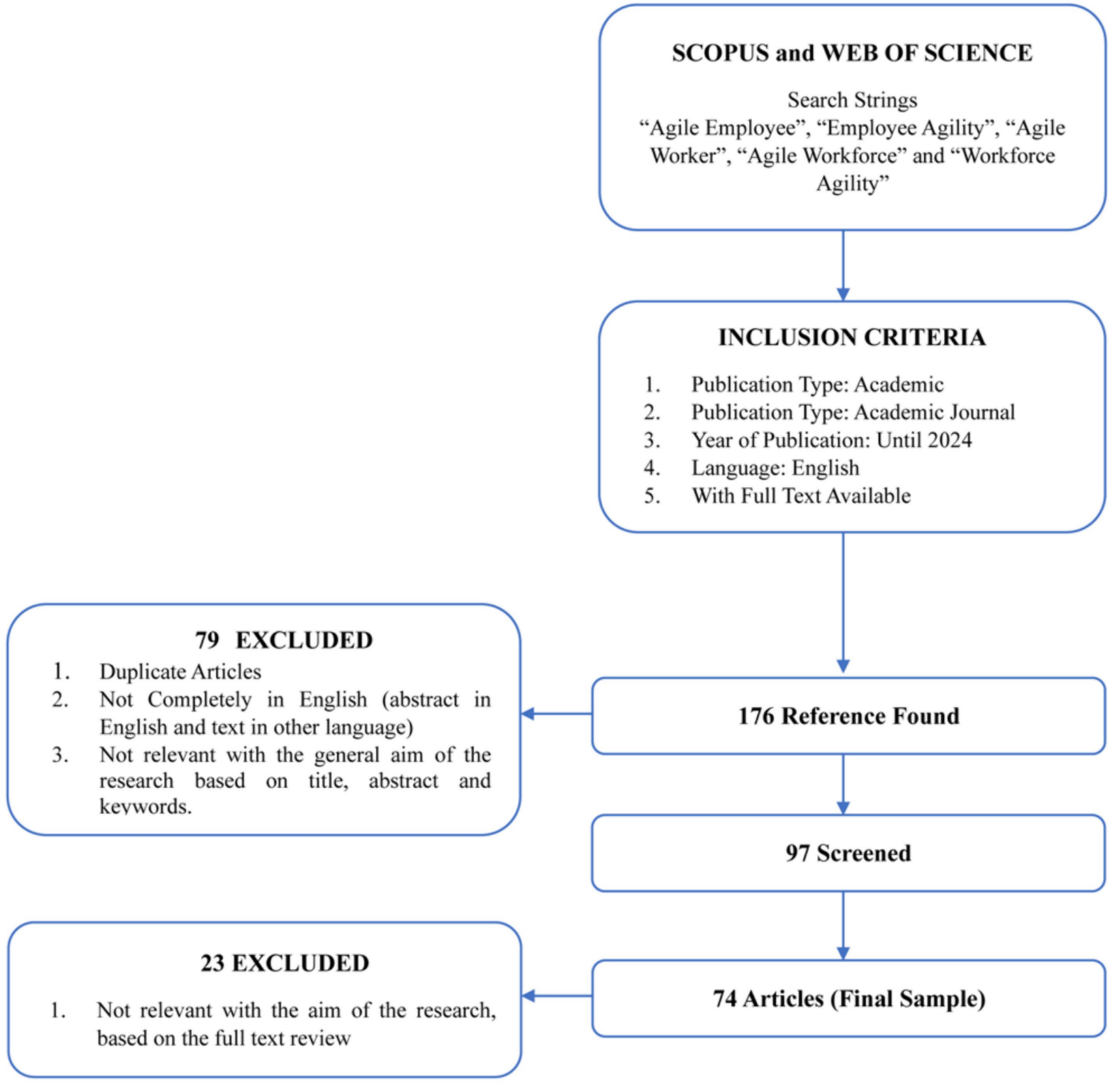
Search strategy. Source: data processed.

In conducting the analysis, it was important for us to consider the concept of saturation. Saturation is a criterion for stopping data collection and/or analysis (Saunders et al., 2018). Referring to the concept by Rahimi and Khatooni (2024), we acknowledged the achievement of data saturation as we did not limit the sample size from the time the first article related to workforce agility was published until the present. We ensured that no new data were obtained up until the year 2024. We assessed that the sample was sufficient to answer the research questions and that all samples had been investigated. The collected data effectively demonstrated diversity, depth, and differences to confirm content validity. We achieved saturation through prolonged engagement, continuous observation, and in-depth description strategies (Rahimi and Khatooni, 2024) (Figure 1).

## 3 Results

In this segment, as outlined in the preceding section, a detailed examination of the outcomes was provided.

### 3.1 Position of the workforce agility variable

This study classifies the workforce agility variable into four roles. First, as an independent variable, workforce agility appears 18 times. In this condition, workforce agility is a variable whose values influence other variables (Andrade, 2021). An example is the study by Franco and Landini (2022) identifying how workforce agility affects innovative performance. Second, as a dependent variable (influenced by other factors) (Andrade, 2021), workforce agility was examined by 35 studies. For example, the research by Talwar et al. (2023) titled “How social networking ties mediate the associations between enterprise social media affordances and employee agility?” positions employee agility as the outcome variable influenced by enterprise social media affordances. Furthermore, the mediating variable was identified to explain the types and impacts of relationships between independent and dependent variables to more accurately and functionally determine the nature of these variables (Namazi and Namazi, 2016). Workforce agility as a mediating variable has been reviewed by eight researchers, including Rasheed et al. (2023), Das et al. (2022), Srivastava and Gupta (2022), Sameer (2022), Herlina et al. (2021), Varshney and Varshney (2020), Raut et al. (2022) and Jannah (2021). Lastly, as a moderating variable, workforce agility enhances research design to produce more realistic and accurate findings by influencing (strengthening and weakening) the relationship between independent and dependent variables (Namazi and Namazi, 2016). The role of workforce agility as a moderating variable can be found in the empirical studies of Braun et al. (2017) and Jin et al. (2020). Table 1 summarizes the studies on workforce agility in these four roles.

**Table 1:** Frequencies of workforce agility position variables.

**Variable position**	**Frequency**	**Research**
Independent	18	Al Hammouri et al. (2023), Heidt et al. (2023), Vuckovic et al. (2023), Ajgaonkar et al. (2022), Durst et al. (2023), Franco and Landini (2022), Saleem et al. (2021), Abrishamkar et al. (2021), Thayyib and Khan (2021), Park and Park (2021), Salmen and Festing (2022), Salih and Almahmeed (2021), Alavi (2016), Al-Faouri et al. (2014), Sohrabi et al. (2014), Qin and Nembhard (2015), Muduli and Choudhury (2024), and Zhang et al. (2022)
Mediating	8	Rasheed et al. (2023), Das et al. (2022), Srivastava and Gupta (2022), Sameer (2022), Herlina et al. (2021), Varshney and Varshney (2020), Raut et al. (2022), and Jannah (2021)
Moderating	2	Braun et al. (2017) and Jin et al. (2020)
Dependent	35	Talwar et al. (2023), Hanu et al. (2023), Zhang et al. (2022), Zandi et al. (2022), Pitafi and Ren (2021), Lai et al. (2021), Zhu et al. (2021), Pitafi et al. (2020), Wei et al. (2020), Doeze Jager-van Vliet et al. (2019), Pitafi et al. (2019), Pitafi et al. (2018), Sherehiy et al. (2007), Maran et al. (2022), Saeed et al. (2022), Lim et al. (2021), Soliman et al. (2021), Müceldili et al. (2020), Munteanu et al. (2020), Storme et al. (2020), Paul et al. (2020), Menon and Suresh (2020), Patil and Suresh (2019), Goswami and Kumar (2018), Muduli and Pandya (2018), Muduli and Pandya (2018), Muduli (2017), Muduli (2016), Alavi (2016), Alavi et al. (2014), Sherehiy and Karwowski (2014), Sumukadas and Sawhney (2004), Naim et al. (2023), Sun et al. (2023), Cyfert et al. (2022), and Pitafi (2024)

### 3.2 Identification of analysis unit

The analysis unit refers to the part or element analyzed in a study. In social research, the analysis units broadly include individual and organizational levels. Regarding the individual level, workforce agility has been reviewed 54 times until 2024 by various researchers, such as Leask and Ruggunan (2021), Al Hammouri et al. (2023), and Talwar et al. (2023). On the other hand, at the organizational level, workforce agility has been examined by only ten researchers, including Das et al. (2022), Franco and Landini (2022), Abrishamkar et al. (2021), Goswami and Kumar (2018), Alavi (2016), Alavi et al. (2014), Qin and Nembhard (2015), Tamtam and Tourabi (2020), Breu et al. (2002), and Pitafi (2024). Other researchers like Sherehiy and Karwowski (2014), Tessarini Junior and Saltorato (2021), Janani and Vijayalakshmi (2023), Muduli and Choudhury (2024), and Richter et al. (2018) have utilized literature (artifacts) as an analytical unit to identify workforce agility. Table 2 shows the studies on workforce agility using the three different analysis units.

**Table 2:** Frequency of analysis units.

Unit of analysis	Frequency	Research
Individual	59	Leask and Ruggunan (2021), Al Hammouri et al. (2023), Talwar et al. (2023), Rasheed et al. (2023), Heidt et al. (2023), Hanu et al. (2023), Zhang et al. (2022), Zandi et al. (2022), Pitafi and Ren (2021), Lai et al. (2021), Zhu et al. (2021), Pitafi et al. (2020), Jin et al. (2020), Wei et al. (2020), Doeze Jager-van Vliet et al. (2019), Pitafi et al. (2019), Pitafi et al. (2018), Braun et al. (2017), Vuckovic et al. (2023), Petermann and Zacher (2022), Srivastava and Gupta (2022), Franco and Landini (2022), Maran et al. (2022), Saeed et al. (2022), Sameer (2022), Saleem et al. (2021), Lim et al. (2021), Thayyib and Khan (2021), Soliman et al. (2021), Herlina et al. (2021), Müceldili et al. (2020), Varshney and Varshney (2020), Munteanu et al. (2020), Muduli and Pandya (2018), Muduli and Pandya (2018), Muduli (2017), Muduli (2016), Al-Faouri et al. (2014), Sohrabi et al. (2014), Sumukadas and Sawhney (2004), Storme et al. (2020), Sherehiy et al. (2007), Menon and Suresh (2022), Ajgaonkar et al. (2022), Durst et al. (2023), Salmen and Festing (2022), Patil and Suresh (2019), Paul et al. (2020), Menon and Suresh (2020), Chonko and Jones (2005), Hopp and Van Oyen (2004), Oyen et al. (2001), Salih and Almahmeed (2021), Petermann and Zacher (2022), Park and Park (2021), Raut et al. (2022), Naim et al. (2023), Sun et al. (2023), Cyfert et al. (2022), Zhang et al. (2022), and Jannah (2021)
Organizational	10	Das et al. (2022), Franco and Landini (2022), Abrishamkar et al. (2021), Goswami and Kumar (2018), Alavi (2016), Alavi et al. (2014), Qin and Nembhard (2015), Tamtam and Tourabi (2020), Breu et al. (2002), and Pitafi (2024)
Artifact	5	Sherehiy and Karwowski (2014), Tessarini Junior and Saltorato (2021), Janani and Vijayalakshmi (2023), Muduli and Choudhury (2024), and Richter et al. (2018)

### 3.3 The purpose of research publication

The objectives regarding the identified focus of the publication needed further exploration. The articles were categorized into three objectives: expanding/linking theory, developing theory, and review/summary. The results showed that the primary purpose of workforce agility research was to expand and link theory (63; 85.14%), which is consistent with the idea that the field was in the initial research stage, introducing or elaborating concepts. The limited number of publications aiming to develop new theory (3; 4.05%) was not surprising, given the difficulty of creating an original conceptual framework. Examples of articles in the category of developing new theory included Braun et al. (2017), Menon and Suresh (2022), Petermann and Zacher (2022), and Braun et al. (2017) focused on developing, validating, and practically implementing a scale measuring employee agility and resilience as part of a program to support an alternative method of managing organizational change. Menon and Suresh (2022) developed a conceptual model for measuring workforce agility in higher education with 30 attributes. Petermann and Zacher developed an inductive taxonomy of workforce agility behaviors consisting of ten dimensions: (1) accepting change, (2) decision-making, (3) creating transparency, (4) collaboration, (5) reflection, (6) user-centricity, (7) iteration, (8) testing, (9) self-organization, and (10) learning. The eight remaining studies (10.81%) aimed to deliver reviews.

### 3.4 Research methodology

The review showed that, out of the total articles (56; 75.68%), one adopted a descriptive analysis method, one applied numerical analysis, and 54 used a cross-sectional method. Cross-sectional research commonly used questionnaires adopted and adapted from Alavi et al. (2014), Braun et al. (2017), Breu et al. (2002), Cai et al. (2018), Cubiks (2014), Dyer and Shafer (2003), Griffin and Hesketh (2003), Gunasekaran (1999), Liu et al. (2015), Muduli (2016, 2017), Petermann and Zacher (2022), Pitafi et al. (2018), Pulakos et al. (2000), Sawhney and Piper (2002), Sherehiy (2008), Sherehiy and Karwowski (2014), Sherehiy et al. (2007), Tamtam and Tourabi (2020), as well as Thayyib and Khan (2021). The collected data were analyzed using various methods such as regression, structural equation modeling, path analysis, hierarchical regression, path analysis, Wilcoxon rank-sum test (grouped), fuzzy logic method, logistic regression, and one-way factorial ANOVA. Furthermore, 18 qualitative studies of various types were identified, including reviews, comparative analysis, cluster analysis, MICMAC analysis, SLR, and descriptive analysis. Data collection methods for this qualitative research comprised literature research, narrative reviews, and interviews. A mixed-methods method was found in one article by Storme et al. (2020), using exploratory and cross-sectional research and interviews and questionnaires as data collection tools. This research constitutes a replication of two previous qualitative systematic literature reviews conducted by Salmen and Festing (2022) and Tessarini Junior and Saltorato (2021). A summary of prior studies on workforce agility with different methods is shown in Table 3. With different research questions, we aim to develop the literature on workforce agility further.

**Table 3:** Frequencies of articles based on research methods and types.

Methodology	Type	Data collection method	Frequency	Research
Quantitative	Descriptive analysis	–	1	Oyen et al. (2001)
	Cross-Sectional	Questionnaire	54	Leask and Ruggunan (2021), Al Hammouri et al. (2023), Talwar et al. (2023), Rasheed et al. (2023), Heidt et al. (2023), Hanu et al. (2023), Zhang et al. (2022), Zandi et al. (2022), Pitafi and Ren (2021), Lai et al. (2021), Zhu et al. (2021), Pitafi et al. (2020), Jin et al. (2020), Wei et al. (2020), Doeze Jager-van Vliet et al. (2019), Pitafi et al. (2019), Pitafi et al. (2019), Pitafi et al. (2018), Braun et al. (2017), Vuckovic et al. (2023), Das et al. (2022), Petermann and Zacher (2022), Srivastava and Gupta (2022), Franco and Landini (2022), Maran et al. (2022), Saeed et al. (2022), Sameer (2022), Saleem et al. (2021), Abrishamkar et al. (2021), Lim et al. (2021), Thayyib and Khan (2021), Soliman et al. (2021), Herlina et al. (2021), Müceldili et al. (2020), Varshney and Varshney (2020), Munteanu et al. (2020), Goswami and Kumar (2018), Muduli and Pandya (2018), Muduli (2017), Muduli (2016), Alavi (2016), Alavi et al. (2014), Al-Faouri et al. (2014), Sohrabi et al. (2014), Sherehiy and Karwowski (2014), Sumukadas and Sawhney (2004), Raut et al. (2022), Naim et al. (2023), Sun et al. (2023), Tamtam and Tourabi (2020), Breu et al. (2002), Cyfert et al. (2022), Zhang et al. (2022), Jannah (2021), and Pitafi (2024)
	Numeric analysis	Literature review	1	Qin and Nembhard (2015)
Qualitative	Review	Literature review	4	Sherehiy et al. (2007), Paul et al. (2020), Menon and Suresh (2020), and Menon and Suresh (2022)
		Interview	1	Ajgaonkar et al. (2022)
	Comparative analysis	Questionnaire	1	Durst et al. (2023)
		Narrative review	3	Park and Park (2021), Chonko and Jones (2005), Hopp and Van Oyen (2004)
	Cluster analysis	Questionnaire	1	Petermann and Zacher (2022)
	MICMAC analysis	Interview and questionnaire	1	Patil and Suresh (2019)
	Systematic literature review	Literature review	5	Salmen and Festing (2022), Tessarini Junior and Saltorato (2021), Janani and Vijayalakshmi (2023), Muduli and Choudhury (2024), and Richter et al. (2018)
	Descriptive analysis	Interview and questionnaire	1	Salih and Almahmeed (2021)
Mix method	Exploration. cross-Sectional	Interview and questionnaire	1	Storme et al. (2020)

### 3.5 Theoretical basis and relationships

Previous researchers have investigated the workforce agility research model using various fundamental theories. We found more than 25 fundamental theories, which we categorized into nine theory categories. These nine theories were reformulated into four general theories: Organizational and Management Theory, Communication and Social Interaction Theory, Behavior and Learning Theory, and Economic Theory.

Organizational and Management Theory consists of three main theories (organization theory, management theory, and psychological theory) and nine fundamental theories (Self-Determination Theory, Dynamic Capability Theory, Resource-Based Theory, Integration of Organizational Support Theory and Organizational Learning Theory, Organizational Evolution Theory, Corporate Growth Theory, Job Demand-Resource Theory, Minnesota Theory of Work Adjustment, and Classical Goal Setting Theory). In organizational and management theory, Self-Determination Theory is the most frequently used theory (five times). Based on the Self-Determination Theory, workforce agility has been found to influence innovation (Franco and Landini, 2022). Additionally, its influence extends to various performance aspects, including innovative performance, task performance, citizenship behavior, fatigue, and job satisfaction (Petermann and Zacher, 2022). Workforce agility also plays a mediating role in the relationship between workplace spirituality and well-being (Srivastava and Gupta, 2022) and the involvement of creative processes in agility performance regarding antecedents, such as cognitive style and job-related curiosity (Müceldili et al., 2020). Dynamic Capability Theory was identified in two studies, explaining how workforce agility shapes supportive factors for remote work related to its implementation success (Heidt et al., 2023). Dynamic Capability Theory also explains that workforce agility is shaped by (1) internal sources, such as skill and competency updates, flexibility and mobility, agile culture, and customer orientation in organizational processes, and (2) external sources, such as resources available in project-based recruitment markets (Ajgaonkar et al., 2022).

Communication and Social Interaction Theory consists of Communication Theory, Social Interaction Theory, Information Processing Theory, and Relationship Theory. Further development leads to Information Processing Theory, Visibility of Communication Theory, Relational Capital Theory, Combining the Conservation of Resources Theory and Social Exchange Theory, Integration of Affordance Theory and Social Network Theory, Integration of Social Exchange Theory, Information Sharing Theory, and Transactional Memory Theory, Integration of Planned Behavior Theory, Social Cognitive Theory, and Unified Theory of Acceptance and Use of Technology, Cognitive Theories, Integration of the Unified Theory of Acceptance and Use of Technology and Social Exchange Theory. Information Processing Theory is a popular theory in Communication and Social Interaction Theory. Related studies highlight various essential roles in understanding factors influencing employee agility. Lai et al. (2020) found that task independence and task autonomy act as mediators in the influence of IT competence on employee agility. Pitafi et al. (2020) demonstrated that job expertise and IT skills moderate the influence of Corporate Social Media usage on employee agility. Wei et al. (2020) highlight the mediating role of meta-knowledge and the moderating role of digital skills in the relationship between MSP usage and employee agility. Pitafi et al. (2019) found that communication quality acts as a mediator in the influence of MSP usage and psychological safety on employee agility. Lastly, Pitafi et al. (2018) highlighted the moderating role of MSP usage in the relationship conflict on employee agility. These results demonstrate the complexity and relevance of various factors in understanding the dynamics of employee agility in modern organizational contexts.

Behavior and Learning Theory includes the Integration of Planned Behavior Theory, Social Cognitive Theory, Unified Theory of Acceptance and Use of Technology, Spillover Theory, and Integration of Social Exchange Theory, Information Sharing Theory, and Transactional Memory Theory, which are new concepts that have been less explored. Finally, Economic Theory involves Pricing Theory, which asserts that Workforce Agility is crucial in shaping organizational agility (Qin and Nembhard, 2010).

**Figure 2:**
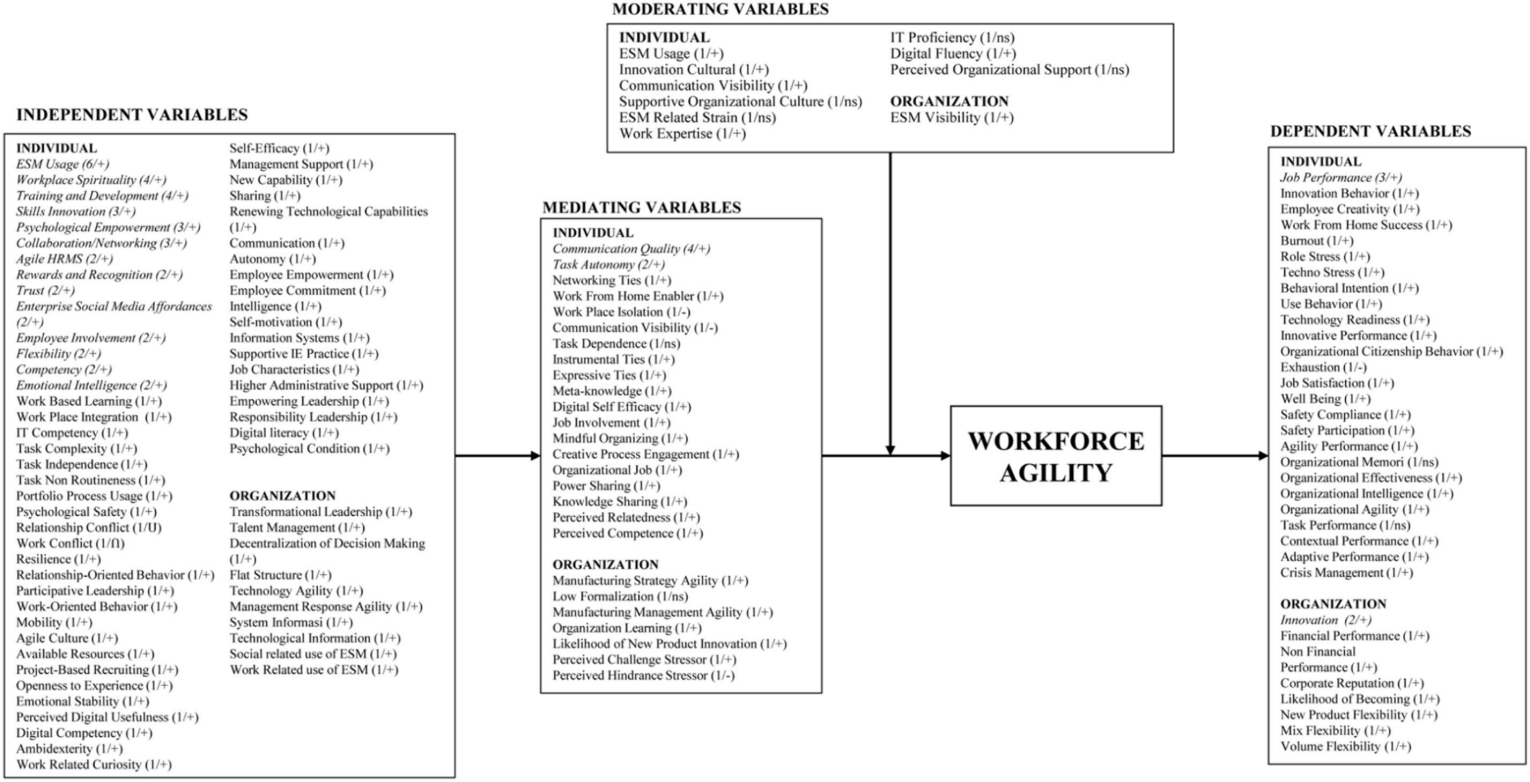
Summary of variables – a nomological network. Source: Data processed. The parentheses contain information about the number of prior studies examining the given variables and the results. Variables in italics have been studied frequently. (+) = positively related; (−) = negatively related; (ns) = non significantly related to workforce agility; ESM, enterprise social media.

In addition to these primary theories, more than 20 other theories were recognized (Table 4), showing a significant diversity in interpreting workforce agility theories. The theories were constructed around various themes, illustrated by the Communication Visibility theory (Pitafi and Ren, 2021; Rasheed et al., 2023), comprising the themes of Communication Visibility and ESM-Related Strain. Similarly, the Unified Theory of Acceptance and Use of Technology and Social Exchange Theory were associated with various themes, such as organizational support, digitalization benefits, and task performance in blended working (Sameer, 2022). For mapping the landscape of existing research on workforce agility, this study explores the relationships between workforce agility and its antecedents, outcomes, moderators, or mediators, culminating in a comprehensive summary of variables in a nomological network. It can be illustrated in Figure 2 as follows:

**Table 4:** Theories explaining the relationship between workforce agility and its outcomes.

Theory			Freq	Examples of supported relationships in literature
First theory	Second theory	Third theory		
Organizational and management theory	Organization theory Management theory Psychological theory	Self-determination theory	5	Workforce agility influences product innovation and process innovation (Franco and Landini, 2022)
				The impact of workforce agility on innovative performance, task performance, organizational citizenship, exhaustion, and job satisfaction (Petermann and Zacher, 2022)
				The mediating role of workforce agility in the influence of workplace spirituality on well-being (Srivastava and Gupta, 2022)
				The mediating role of creative process engagement in the influence of antecedents (cognitive style) and work-related curiosity on agility performance (Müceldili et al., 2020)
				All four ESM affordances contribute to perceived relatedness and perceived competence; visibility and association affordances also have positive impacts on perceived autonomy; and all three psychological needs satisfaction positively impact employee agility (Sun et al., 2023)
		Dynamic capability theory	2	Workforce agility is shaped by (1) internal sources such as skill and competency updates, flexibility and mobility, an agile culture, and customer orientation within organizational processes; (2) External sources, including resources available in the project-based recruitment market (Ajgaonkar et al., 2022)
				The mediating role of work-from-home enablers in the influence of agile work on work-from-home success (Heidt et al., 2023)
		Resource based theory	2	The mediating role of organizational learning in the influence of low formalization, decentralization of decision-making, and flat structure on workforce agility (Alavi et al., 2014)
				The characteristics, capabilities, and agile behaviors play a crucial role in driving the digital transformation process (Muduli and Choudhury, 2024)
		Integration of organizational support theory and organizational learning theory	1	The moderating role of a supportive organizational culture in the influence of work-based learning on employee agility (Hanu et al., 2023)
		Organizational evolution theory	1	Workforce agility influences organizational memory (Al-Faouri et al., 2014)
		Corporate growth theory	1	The mediating role of the likelihood of new product innovation in the influence of workforce agility on the probability of becoming a high-growth firm (Abrishamkar et al., 2021).
		Job demand-resource theory	1	The moderating role of leader-member exchange quality and teacher agility in the influence of role stress and techno stress on burnout (Jin et al., 2020)
		Minnesota theory of work adjustment	1	Relationship-oriented behavior, participative leadership, and job-oriented behavior influence workforce agility (Sherehiy et al., 2007)
		Classical goal-setting theory	1	The use of portfolio processes supports employee agility (Doeze Jager-van Vliet et al., 2019)
Communication and social interaction theory	Communication theory Social interaction theory Information processing theory Relationship theory	Information Processing Theory	5	The mediating role of task independence and task autonomy in the influence of IT competency on employee agility (Lai et al., 2021)
				The moderating role of work expertise and IT proficiency in the influence of Enterprise Social Media (ESM) usage on employee agility (Pitafi et al., 2020)
				The mediating role of meta-knowledge and the moderating role of digital fluency in the influence of Enterprise Social Media (ESM) usage on employee agility (Wei et al., 2020)
				The mediating role of communication quality in the influence of Enterprise Social Media (ESM) usage and psychological safety on employee agility (Pitafi et al., 2019)
				The moderating role of Enterprise Social Media (ESM) usage in workplace relationship conflict on employee agility (Pitafi et al., 2018)
		The visibility of communication theory	3	The mediating role of workforce agility and the moderating role of communication visibility in the influence of Enterprise Social Media (ESM) usage on employee creativity (Rasheed et al., 2023)
				The mediating roles of communication quality and communication visibility, along with the moderating role of ESM-related strain, in the effect of ESM usage on employee agility (Pitafi and Ren, 2021)
				Using ESM (social and work-related) negatively affects job challenge stress and hindrance stress. Job challenge stress has a significant positive impact on employee agility, while hindrance stress correlates negatively with it. Visibility of ESM enhances the relationship between job challenge stress and employee agility, but it does not have a significant moderating impact on the relationship between hindrance stress and employee agility (Pitafi, 2024)
		Relational capital theory	2	The mediating role of communication quality and trust in the influence of the benefits of using corporate social media on employee agility (Zhang et al., 2022)
				ESMU is positively related to communication quality and trust; innovation culture positively moderates ESMU and employee agility; and high communication quality and trust can result in high agility. However, innovation culture does not have a significant moderating effect on ESMU and communication quality (Zhang et al., 2022)
		Combining the conservation of resources theory and social exchange theory	1	Empowering leadership contributes to psychological safety at the workplace, promoting employees’ knowledge-sharing behavior and leading to employee agility (Naim et al., 2023)
		Integration of affordance theory and social network theory	1	The role of mediating networking ties in the relationship between enterprise social media affordances and workforce agility (Talwar et al., 2023)
		Integration of social exchange theory, information sharing theory, and transactional memory theory	1	The mediating role of workplace isolation in the influence of enterprise social media and workplace integration on employee agility (Zandi et al., 2022)
		Integration of planned behavior theory, social cognitive theory, and unified theory of acceptance and use of technology	1	Workforce agility contributes to the formation of behavioral intention and use behavior (Vuckovic et al., 2023)
		Cognitive theories (self-determination theory, job characteristics theory, and sensemaking theory)	1	Psychological empowerment influences workforce agility (Muduli and Pandya, 2018)
		Integration of the unified theory of acceptance and use of technology and social exchange theory	1	The mediating role of workforce agility and the moderating role of perceived organizational support in the influence of perceived benefits of digitalization on task performance in blended working (Sameer, 2022)
Behavior and learning theory	Behavior and learning theory	Integration of planned behavior theory, social cognitive theory, and unified theory of acceptance and use of technology	1	Workforce agility contributes to the formation of behavioral intention and use behavior (Vuckovic et al., 2023)
		Spillover theory	1	The mediating role of job engagement in the influence of work spirituality on workforce agility and work performance (Saeed et al., 2022)
		Integration of social exchange theory, information sharing theory, and transactional memory theory	1	The mediating role of workplace isolation in the influence of enterprise social media and workplace integration on employee agility (Zandi et al., 2022)
Economic Theory	Economic Theory	Pricing theory	1	Workforce agility plays a crucial role in shaping organizational agility (Qin and Nembhard, 2010)

The contribution to the understanding of workforce agility includes an examination of variables through a nomological network and the presentation of theories underlying these relationships. The identified variables in the literature are at two levels: individual employees (e.g., training and development, skills innovation, psychological empowerment, competency, emotional intelligence, work-based learning, workplace integration, relationship-oriented behavior, creative process engagement, job involvement) and organizations (e.g., manufacturing strategy agility, manufacturing management agility, organization learning, and the likelihood of new product innovation) (Qin and Nembhard, 2010; Muduli and Pandya, 2018; Petermann and Zacher, 2022; Hanu et al., 2023). This observation shows less attention has been given to the relationship between workforce agility and variables at the organizational level than at the individual level. Concerning dependent, mediating, and moderating variables, it is essential to prioritize causal factors of workforce agility at the team level. According to scholars researching other behaviors, investigating crucial causal factors is essential to provide a strong foundation for developing workforce agility. These causal factors may relate to any of the three levels: individual, team, and organizational.

An interesting observation is that some research (though limited) has focused on the potential negative outcomes of workforce agility for employees (e.g., role stress, techno stress, and burnout) (Jin et al., 2020). This research is crucial to offer a comprehensive view of the potential outcomes of workforce agility, including adverse effects. Another unexplored and should-be-explored question is how being an agile worker affects those around, including colleagues and leaders. Given that agile workers may demand resources from colleagues and leaders for personal use, it can lead to unfavorable outcomes, such as stress and loss of future resources.

### 3.6 Avenues for future research

The identification of recommendations for future research is outlined in Table 5 as follows:

**Table 5:** Avenues for future research.

Researcher	Recommendation	General recommendation
Sun et al. (2023)	Exploring further into various ESM capabilities, considering contextual factors as regulators of ESM influence, employing longitudinal designs and data from diverse sources for more reliable analysis results, and expanding investigations into backgrounds of other cultures.	Conducting longitudinal research
Zhang et al. (2022)	Establishing a longitudinal database to observe the transfer of ESMU influence on employee agility through innovation culture and considering the intensity of corporate social media usage.	
Pitafi (2024)	Utilizing a longitudinal design, considering the impact of excessive corporate social media use, and expanding the research focus to examine the specific impacts of various ESM platforms. Moreover, it is crucial to involve respondents from diverse countries.	
Saleem et al. (2021)	Stimulating a longitudinal method to address limitations and explore the mediating effects in a broader context.	
Zhu et al. (2021)	Using a longitudinal design and samples from various sources, including interviews with supervisors, to enhance the reliability of conclusions about employee agility, exploring the impact of organizational climate, job stress, and task characteristics, as well as evaluating the feasibility of the Experience Sampling Method (ESM) such as associations, perseverance, and editing ability.	
Rasheed et al. (2023)	Leveraging longitudinal surveys or experimental designs for valid outcomes.	
Jin et al. (2020)	Identifying the dynamic impact of teacher agility in alleviating stress and work fatigue through longitudinal research, obtaining data on teachers experiencing high levels of security stress, and expanding the sample pool for a more comprehensive analysis.	
Doeze Jager-van Vliet et al. (2019)	Longitudinal research on the dynamic impact of teacher agility in alleviating stress and work fatigue should be followed up with a large sample size.	
Das et al. (2022)	Conducting longitudinal research to explore differences among categories of companies.	
Storme et al. (2020)	Using a longitudinal method or cross-panel analysis to investigate the relative contributions of cognitive and emotional factors to workforce agility and providing a more comprehensive picture of individual resources that support agility for organizations.	
Hanu et al. (2023)	Integrating mixed methods, applying a longitudinal method, testing additional moderators, and extending the research to a global context.	Integrating mix method
Park and Park (2021)	Understanding the relationship between adaptive and proactive performance and incorporating perspectives from employees across various industries in a mixed-methods research.	
Jannah (2021)	Using balanced sampling from each industrial sector for a more valid comparison.	Considering the Global-Culture Context
Alavi and Wahab (2017)	Testing the framework in large manufacturing companies, considering performance measurements, and incorporating other aspects of agility.	
Lai et al. (2021)	Applying a similar theoretical model across various countries, assessing the relevance of the structure within the research context, expanding the sample, exploring moderator variations, task environment analysis, testing intervening variables to enhance understanding of employee agility improvement, and expanding similar ideas to the group level for further insights.	
Soliman et al. (2021)	Expanding the sample to various academic and industry fields, integrating a qualitative method, incorporating mediating and moderating variables, and exploring other consequences of Workplace Spirituality (WPS) in higher education, such as faculty loyalty and turnover.	
Durst et al. (2023)	Considering outcome variables, individual characteristics, and pathways at various stages of a crisis to enhance organizational and technological readiness and evaluating methods in diverse institutional and socio-cultural contexts.	
Müceldili et al. (2020)	Conducting longitudinal research and expanding research to various cultural contexts.	
Muduli and Pandya (2018)	Expanding the global scope, adopting a case-based method, empirically testing the antecedents of psychological empowerment, and considering cultural perspectives to understand the impact of social factors on feelings of empowerment and agile behavior within organizations.	
Ajgaonkar et al. (2022)	Conducting longitudinal quantitative research in various organizations and contexts, with an international focus and a specific emphasis on Small and Medium Enterprises (SMEs) in the UK.	
Al-Faouri et al. (2014)	Improvements in research instruments, inclusion of diverse sectors and countries, exploration of mediating factors, and enhancing the understanding of the impact of workforce agility on organizational learning should be undertaken while expanding the geographical coverage.	
Saeed et al. (2022)	Expanding the sample and geographic coverage, employing different research methods, exploring mediating factors, and balancing participation of both males and females for a more in-depth understanding of spirituality in the workplace and employee agility in dynamic work environments.	Expanding Samples
Goswami and Kumar (2018)	Broadening the scope by examining the relationships between attributes and other dimensions for a more in-depth understanding of the factors influencing the overall performance of Small and Medium Enterprises (SMEs).	
Zandi et al. (2022)	Including the telecommunications sector, banking sector, and developed countries to broaden the scope.	
Sameer (2022)	Considering a longitudinal research design, exploring sectors other than the public sector, addressing low response rates, and investigating the impact of workforce agility related to individual factors, job design, as well as interactions in the context of blended working to provide in-depth insights into the continually evolving work environment.	
Sumukadas and Sawhney (2004)	Testing the theory more rigorously with a large and diverse sample, considering other contextual factors, and developing a measurement scale for workforce agility to understand the impact of team-based production incentives.	
Menon and Suresh (2022)	Testing a conceptual model of workforce agility in various higher education institutions to expand the triggers and attributes while broadening the sample.	
Al Hammouri et al. (2023)	Investigating the impact of Agile HRMS in the government sector, particularly in talent acquisition, learning and development, and employee engagement to understand its influence on organizational performance and provide valuable insights for government agencies in optimizing their performance and effectiveness.	
Pitafi and Ren (2021)	Focusing on the impact of ESM platforms on individual performance with a large sample, considering the work context and country, and including the workforce with minimal education.	
Oyen et al. (2001)	Examining the potential for workforce agility in diverse production environments.	
Herlina et al. (2021)	Increasing the number of respondents, exploring additional factors such as leadership and organizational culture, and potentially reusing the research instrument to investigate ambidexterity as a supporting factor for organizational effectiveness in various research contexts.	Development conceptual model
Janani and Vijayalakshmi, 2023	Empirical testing factors and practices influencing Workforce Resilience, evaluating the proportion of capacity built by each art form, researching other psychological effects of changes such as stress and fatigue, and exploring potential boundary conditions affecting the studied phenomena.	
Muduli and Choudhury (2024)	Conducting further research on critical yet underexplored employee attributes in the context of technology adoption, exploring agile attitude characteristics influencing individuals’ perceptions of technology, and utilizing the tripartite attitude model to investigate the comprehensive impact of agile attitude characteristics on technology acceptance.	
Das et al. (2022)	Testing other mediators such as individual innovation, group innovation, and organizational learning in the context of transformational leadership, talent management, for-profit (FP), not-for-profit (NFP), and corporate responsibility (CR).	
Breu et al. (2002)	Continuing with an inductive strategy to discover additional variables relevant to workforce agility and developing causal models of agility variables.	
Vuckovic et al. (2023)	Incorporating individual factors and the same methods for assumption confirmation provides a foundation for stakeholders to make improvements.	
Braun et al. (2017)	Developing individual resilience through mindfulness and other strategies and examining the impact on business indicators such as absenteeism and adaptability to change.	
Petermann and Zacher (2022)	Considering the formative nature of agility and investigating the mediation and relationship between agility and innovation, as well as training and interventions.	
Salmen and Festing (2022)	Developing steps following the scientific process, such as Hinkin (1998), for researching empirical contextual study on the role of HRM systems and practices in enhancing employee agility, using additional data sources, and designing a conceptual framework with deep theoretical considerations, in addition to Business Source Complete.	
Muduli (2017)	Designing research and organizational practices that prioritize understanding and fostering workforce agility.	
Goswami and Kumar (2018)	Broadening the scope by examining the relationships between attributes and other dimensions for a more in-depth understanding of the factors influencing the overall performance of Small and Medium Enterprises (SMEs).	
Alavi et al. (2014)	Focusing on implementing organic structural methods and organizational learning with system dynamics while investigating the nonlinear relationship between low formalization, organizational learning, and workforce agility.	
Müceldili et al. (2020)	Exploring the underlying factors of job-related curiosity and its impact on job satisfaction, organizational commitment, identification, and psychological well-being.	
Pitafi et al. (2019)	Expanding research by exploring the relationship between employee agility and other performance factors such as training, motivation, and job demands.	
Cyfert et al. (2022)	Further exploring the relationship between psychological empowerment and workforce agility using a more comprehensive measurement scale.	
Rasheed et al. (2023)	Exploring the organizational impact of ESM usage, including employee knowledge-sharing behavior and the managerial role in creativity.	
Abrishamkar et al. (2021)	Investigating whether the dimensions of workforce agility mediate the relationship between new product innovation and other types of innovation and HFG (High-Performance Growth).	
Richter et al. (2018)	Researching the future agile workforce with a primary focus both on organizations and scientific researchers.	
Pitafi et al. (2020)	Exploring additional factors influencing employee agility, expanding the model to understand their impact on performance and turnover intentions, investigating the mediating mechanisms of the Experience Sampling Method (ESM) and the impact on team outcomes, including creativity, performance, conflict, and team environment.	
Srivastava and Gupta (2022)	Extending the sample to all of India, exploring negative variables, and incorporating mediation through personality traits and comparative analysis to gain profound insights into the relationship between spirituality and workplace well-being.	
Maran et al. (2022)	Considering the conformance of interests, organizational context, culture, and company age, proposing alternative personality measures, such as the HEXACO model, and suggesting the need to mitigate technological bias in data collection to deepen the understanding of digital self-efficacy and workplace agility in diverse contexts.	
Salih and Almahmeed (2021)	Testing the high credibility attributes of Work from Anywhere (WFA), expanding the research, and focusing on enhancing workforce performance and achieving excellence to improve overall performance.	
Munteanu et al. (2020)	Exploring the relationship between management styles and workforce flexibility across various company types.	
Muduli (2016)	Exploring the mediating role of variables such as satisfaction, commitment, and trust in the relationship between organizational practices and workforce agility.	
Talwar et al. (2023)	Considering affordability and additional impact, as well as expanding literature by exploring intervention mechanisms, such as mediation variables (organizational and relational factors) and moderation variables (network centrality, network size, frequency of ESM use, active vs. passive usage). Understanding the transmission of the impact of ESM capabilities on agility is critical.	Exploring mediating and moderating variables
Heidt et al. (2023)	Enhancing self-efficacy and employee locus of control in Human Resource Management (HRM) development to support the success of remote work, with organizational support as a key mediator and conducting multi-mediator analysis to understand the complex relationship between flexible work arrangements and future remote work success.	
Pitafi et al. (2019)	Testing different mediating variables and extending research to the team level.	
Tamtam and Tourabi (2020)	Elaborating the theoretical model of Breu et al. (2002) regarding workforce agility and developing a more comprehensive assessment questionnaire. Additionally, it is suggested that cross-organizational case studies be conducted to delve deeper into the factors influencing workforce agility.	Development of work force agility theory
Sherehiy et al. (2007)	Expanding the metrics and framework of agility in research, including literature on employee agility. A theoretical foundation is required to identify the implications of agile management on workforce characteristics by establishing concepts and indicators of agility.	
Thayyib and Khan (2021)	Considering more detailed measurements of workforce agility.	
Franco and Landini (2022)	Identifying data limitations and recommending future research with more robust designs.	The efficient use of evaluation methods
Tessarini Junior and Saltorato (2021)	Expanding data sources by searching for research in other databases and considering subjective aspects in selecting data sources.	
Lim et al. (2021)	Incorporating the restructuring of demographic profiles, using the Partial Least Squares-Multi Group Analysis (PLS-MGA) method to assess differences between groups, exploring comparisons with different populations, and applying similar models in other government departments.	
Muduli (2016)	Expanding the sample and variations and validating through interviews.	
Qin and Nembhard (2010)	Considering additional sources of uncertainty, such as technological risks, and integrating quantitative and qualitative methodologies in understanding workforce agility.	
Hopp and Van Oyen (2004)	Developing a microeconomic framework to conceptualize further and advance this idea.	
Raut et al. (2022)	Gathering data from departments as the unit of analysis can strengthen research findings because crisis management primarily involves teamwork. Additionally, it is essential to consider social responses in the crisis.	The Implementation of a Multilevel Method
Naim et al. (2023)	Conducting tests using multilevel analysis to consider the role of leadership as a group-level variable.	
Pitafi et al. (2019)	Testing different mediating variables and extending research to the team level.	
Pitafi et al. (2018)	Testing the influence of job characteristics such as job demands, autonomy, and supervisor support on employee agility. A larger sample and multi-level research can also be adopted to explore the impact of ESM usage at the team level.	
Park and Park (2021)	Considering cognitive flexibility in the adaptability of individuals and groups to environmental changes.	
Jannah (2021)	Further research at the organizational level is needed to evaluate overall organizational agility.	
Wei et al. (2020)	Evaluating employee agility from a managerial perspective and exploring the concept of team-level employee agility.	

Previous researchers have offered various recommendations as a roadmap for future researchers (Table 5). Future researchers are recommended to conduct longitudinal research, adopt mixed methods, consider global-cultural contexts, expand samples, develop conceptual models, explore mediation and moderation variables, develop workforce agility theory, use more efficient methods, and implement multilevel methods.

1. Conducting Longitudinal Research. Sun et al. (2023), Zhang et al. (2022), Pitafi (2024), Saleem et al. (2021), Rasheed et al. (2023), Jin et al. (2020), Doeze Jager-van Vliet et al. (2019), Lim et al. (2021), Das et al. (2022), Storme et al. (2020), and Zhu et al. (2021) emphasized the importance of utilizing a longitudinal approach to enhance the validity and reliability of the analysis results.
2. Integrating Mixed Methods. Hanu et al. (2023) and Park and Park (2021) underscored the importance of integrating mixed methods to gain a more comprehensive understanding of the phenomena under investigation.
3. Considering Global-Culture Context. Jannah (2021), Ajgaonkar et al. (2022), Alavi and Wahab (2017), and Lai et al. (2021) indicated the need to consider the global context and cultural variations to obtain a more holistic insight. Similarly, Soliman et al. (2021), Durst et al. (2023), Müceldili et al. (2020), and Muduli and Pandya (2018) emphasized the importance of considering the local and cultural context by expanding the research sample across various industrial fields and organizational environments.
4. Expanding Samples. Saeed et al. (2022), Goswami and Kumar (2018), Zandi et al. (2022), Sameer (2022), Sumukadas and Sawhney (2004), Al Hammouri et al. (2023), and Pitafi and Ren (2021) highlighted the importance of expanding the research sample across various sectors and contexts, as well as involving respondents from different countries to gain a more holistic insight.
5. Developing Conceptual Models. Herlina et al. (2021), Janani and Vijayalakshmi, 2023, Muduli and Choudhury (2024), Das et al. (2022), Breu et al. (2002), Vuckovic et al. (2023), Petermann and Zacher (2022), Salmen and Festing (2022), Muduli (2017), Goswami and Kumar (2018), Richter et al. (2018), Pitafi et al. (2020), and Alavi et al. (2014) emphasized the importance of developing, understanding, and evaluating comprehensive models and theories underlying research to understand phenomena. Braun et al. (2017) added that the development of theories and concepts should be tested with careful empirical research.
6. Exploring Mediating and Moderating Variables. Talwar et al. (2023) and Heidt et al. (2023) emphasized the importance of exploring mediating and moderating variables, including multi-mediator analysis, to understand more complex relationships between variables. Meanwhile, Pitafi et al. (2019) recommended testing various mediating variables to broaden understanding of employee agility.
7. Development of Workforce Agility Theory. Tamtam and Tourabi (2020), Sherehiy et al. (2007), and Thayyib and Khan (2021) stressed the importance of developing robust research instruments to measure relevant variables accurately.
8. The Efficient Use of Evaluation Methods. Franco and Landini (2022), Tessarini Junior and Saltorato (2021), Lim et al. (2021), Muduli (2016), and Qin and Nembhard (2010) highlighted the importance of using multi-method approaches, including both quantitative and qualitative data, to gain a more comprehensive understanding of employee agility.
9. The Implementation of a Multilevel Method. Raut et al. (2022), Park and Park (2021), Naim et al. (2023), Pitafi et al. (2019), Jannah (2021), Lim et al. (2021), and Wei et al. (2020) advocated for the use of multi-level methods in their research to obtain a more comprehensive understanding of employee agility. Meanwhile, Pitafi et al. (2018) recommended using multi-level research to explore the impact of enterprise social media (ESM) usage at the team level.

## 4 Discussion

### 4.1 Theoretical framework explaining workforce agility

We have identified 25 theoretical/conceptual frameworks (Table 4), which can be categorized into four groups based on the nature or primary focus of the theory, namely:

#### 4.1.1 Organizational and management theory

Organizational and Management Theory focuses on how organizations can be developed, strengthened, and sustained by maximizing employee potential for long-term success in a competitive, dynamic, and constantly changing environment. The management and organizational theories guiding studies related to workforce agility include nine theories.

1. Self-Determination Theory. It details intrinsic and extrinsic motivation by fulfilling basic psychological needs, namely workplace spirituality supporting workforce agility, demonstrating well-being, innovative performance, task performance, organizational citizenship, and job satisfaction (Petermann and Zacher, 2022).
2. Dynamic Capability Theory, where workforce agility is shaped by internal resources such as skill and competency updates, flexibility and mobility, agile culture, and customer orientation in organizational processes (Ajgaonkar et al., 2022).
3. Resource-Based Theory, examining the role of organizational learning, low formalization, decentralization of decision-making, and flat structures on workforce agility (Alavi et al., 2014).
4. The Integration of Organizational Support Theory and Organizational Learning Theory, where a supportive organizational culture enhances work-based learning to increase employee agility (Hanu et al., 2023).
5. Organizational Evolution Theory, which explains the relationship between workforce agility and the formation of organizational memory (Al-Faouri et al., 2014).
6. Corporate Growth Theory, discussing how workforce agility shapes the potential for new product innovations, eventually resulting in high-growth firms (Abrishamkar et al., 2021).
7. Job Demand Resource (JD-R) Theory, which addresses how job resources and demands affect worker exhaustion and motivation. Jin et al. (2020) explored the moderating role of leader-member exchange quality and teacher agility on stress, utilizing JD-R as a foundation to investigate the dynamic relationship between job demands, resources, and performance (Jin et al., 2020).
8. Minnesota Theory of Work Adjustment, employed by Lim et al. (2021) to expose the moderating role of social media use in enhancing digital competency-based workforce agility, emphasizing the importance of an adaptive and supportive work environment according to TWA principles (Lim et al., 2021).
9. Classical Goal Setting Theory, which sets specific and challenging goals to improve worker performance. Research highlights how the application of portfolio processes supports employee agility, underscoring the importance of clear and attainable goals for performance enhancement (Doeze Jager-van Vliet et al., 2019).

#### 4.1.2 Communication and social interaction theory

Communication and Social Interaction Theory delves into how individuals interact and communicate within social contexts, including organizational and workplace environments. Fundamental aspects of this theory include the influence of social networks, expertise in IT, social exchange, information sharing, and the visibility of communication and social cognition in shaping behaviors and interactions among individuals or groups (Lai et al., 2021; Pitafi and Ren, 2021; Talwar et al., 2023; Vuckovic et al., 2023). Theories classified under Communication and Social Interaction Theory are ten theories.

1. Information Processing Theory, which identifies that employees tend to be more agile and adaptable to changes by having IT expertise and the freedom to execute tasks (Lai et al., 2021).
2. Visibility of Communication Theory, which focuses on how effective and open communication can reduce tensions related to using ESM (enterprise social media) and increase employee responsiveness (Pitafi and Ren, 2021).
3. Relational Capital Theory, which focuses on how people interact in interpersonal relationships, offering a deeper understanding of social dynamics and communication between individuals (Zhang et al., 2022).
4. Conservation of Resources Theory and Social Exchange Theory, which suggests that empowering leadership contributes to psychological safety in the workplace, promotes employees’ knowledge-sharing behavior, and leads to employee agility (Naim et al., 2023).
5. Social Network Theory, which highlights that solid and well-connected networks assist in efficiently disseminating information and enhancing the agility of organizations and individuals. In this context, strong networks act as facilitators, enabling smooth information flow and social support essential in responding to changes (Talwar et al., 2023).
6. Affordance Theory, which focuses more on how environments (both physical and digital) enable or limit certain behaviors, having significant implications for the design of human-computer interaction and media studies (Talwar et al., 2023).
7. Integration of Social Exchange Theory and Information Sharing Theory, which reveals the importance of interaction and productive information exchange in preventing the negative consequences of workplace isolation on employee agility and integration (Zandi et al., 2022).
8. Social Cognitive Theory, which explores how individual behavior (workforce agility) influences behavioral intentions and individual decisions (Vuckovic et al., 2023).
9. Cognitive Theory, which emphasizes the importance of meeting employees’ psychological needs for autonomy, competence, and relatedness to increase their ability to adapt and innovate (Muduli and Pandya, 2018).
10. Unified Theory of Acceptance and Use of Technology by Sameer (2022), which shows that accepting and integrating new technology and organizational support enhance employees’ ability to perform tasks effectively within a flexible work system. It also clarifies that the interaction between individual, psychological, and technology factors has a significant influence on workforce agility and performance in the current digital era (Sameer, 2022).

#### 4.1.3 Behavior and learning theory

The Theory of Planned Behavior, Spillover Theory, and Transactional Memory Theory are classified under the behavior and learning category because each provides a framework for understanding how individuals develop and adjust their behavior based on the influences of the environment, experiences, and interpersonal interactions. The Theory of Planned Behavior emphasizes the importance of workers’ flexibility and adaptability in fostering technology adoption (Vuckovic et al., 2023). Spillover Theory underscores the relationship between emotional well-being, productivity, and adaptability (Saeed et al., 2022). Lastly, the framework of Transactional Memory Theory highlights the impact of social media use and team integration on employees’ flexibility in facing isolation (Zandi et al., 2022). Together, these studies offer insights into how psychosocial factors and technology interactions at work contribute to workforce agility.

#### 4.1.4 Economic theory

Pricing Theory is a part of Economic Theory that explains how the market prices of goods and services are formed through the interaction between demand and supply, along with other factors such as production costs, competition, and consumer preferences. Organizational agility resulting from workforce agility, the ability of companies to quickly and efficiently respond to changes in market conditions, including consumer demand and competitor actions, is a critical factor influencing pricing strategies (Qin and Nembhard, 2010).

The variables related to workforce agility have been identified as four types (Figure 2):

##### 4.1.4.1 Independent variables

The identified independent variables concerning workforce agility amounted to 57, where 50 were analyzed at the individual level and the rest at the organizational level. Most variables were examined once, with only 13 variables reviewed more than once (for example, ESM usage six times; workplace spirituality, training, and development four times; skills innovation, psychological empowerment, collaboration/networking three times each; and agile HRMS, rewards and recognition, enterprise social media affordances, employee involvement, flexibility, competency, and emotional intelligence twice). The majority of research findings indicated significant positive outcomes. However, there are two variables: (1) Relationship conflict, which shows a U-shaped influence on workforce agility, and (2) Work conflict, wherein its interaction with workforce agility forms an inverted U-shape (Ո).

##### 4.1.4.2 Mediating variables

Nineteen variables were explored just once as mediating variables related to workforce agility, such as networking ties, work-from-home enablers, workplace isolation, and communication visibility. Most findings showed significant positive effects, except for workplace isolation and communication visibility, which contributed negatively, and task dependence, which was found to have no significant influence. Two mediating variables, communication quality and psychological empowerment, were analyzed more than once—three times for communication quality and twice for psychological empowerment.

##### 4.1.4.3 Moderating variables

Very few (eight) moderating variables related to workforce agility were examined. Four had a significant positive impact (ESM usage, communication visibility, work expertise, digital fluency), while the rest (IT proficiency, supportive organizational culture, ESM-related strain, perceived organizational support) showed no significant effect.

##### 4.1.4.4 Dependent variables

The dependent variables found in our analysis amounted to 32. Thirty were examined once, the rest were reviewed thrice, and innovation variables were reviewed twice. Most research findings showed significant positive effects, except for exhaustion (negative), organizational memory, and task performance were found insignificant.

### 4.2 The interplay between the variables and theories in explaining workforce agility

With the various findings and diverse theories in our corpus, we compiled the results to identify patterns of workforce agility dynamics. Without intending to discredit other variables and theories used so far, but with the aim of simplicity, we focused on theories and variables that have shown a strong association with workforce agility.

The Communication and Social Interaction Theory was a famous group of theories used to identify the determinants of workforce agility. Based on our research, the Communication and Social Interaction Theory was found in 17 hypothesis tests, with dominance in the Information Processing Theory as its part. Information Processing Theory discusses various variables, such as IT competency, IT proficiency, task independence, task autonomy, meta-knowledge, digital fluency, communication quality, and enterprise social media (ESM) usage. These concepts have a close relationship with workforce agility. Information Processing Theory emphasizes that individuals actively manage and process information in various ways, affecting their ability to adapt and perform in dynamic work environments.

An example is the research on the mediation of task independence and task autonomy in the relationship between IT competency and workforce agility. Lai et al. (2021) highlighted how individuals’ ability to process information through technology could affect their level of independence and autonomy in completing tasks, which could affect their agility. Also, there is research on the moderation of work expertise and IT proficiency in the relationship between ESM usage and workforce agility. Pitafi et al. (2020) suggested that levels of job expertise and technological skills could moderate the effects of using enterprise social media on employee agility. Information Processing Theory provided a helpful framework for understanding how information and technology-related factors affected workforce agility, which could help organizations optimize their employees’ performance and adaptability in the digital era (Pitafi et al., 2018, 2019, 2020; Wei et al., 2020; Lai et al., 2021).

Furthermore, researchers have also paid attention to management and organizational theory. This theory was traced 15 times, focusing on the Self-Determination Theory. Self-Determination Theory discusses various variables related to workforce agility. This theory highlights the importance of psychological factors underlying individuals’ intrinsic motivation to achieve well-being and optimal performance. Franco and Landini (2022) found that workforce agility influenced product and process innovation, aligning with Self-Determination Theory (SDT), which emphasizes the importance of autonomy, competence, and relatedness in motivating individuals to innovate. Petermann and Zacher (2022) showed that workforce agility affected innovative performance, task performance, organizational citizenship, burnout, and job satisfaction, also within the SDT framework that links basic needs with employee performance and well-being. Srivastava and Gupta (2022) asserted that workforce agility mediated the impact of workplace spirituality on well-being, demonstrating how connection with spiritual values can enhance well-being through the fulfillment of psychological needs. Sun et al. (2023) found that the four uses of ESM enhanced perceptions of relatedness and competence, fulfilled psychological needs, and increased employee agility. SDT provides a framework for understanding how intrinsic motivational factors influence workforce agility, helping organizations improve employee performance and well-being (Franco and Landini, 2022; Petermann and Zacher, 2022; Srivastava and Gupta, 2022; Sun et al., 2023). These findings reveal that Communication and Social Interaction Theory and Management and Organization Theory offer a more comprehensive explanation of the mechanisms and processes created or resulting from workforce agility.

**Figure 3:**
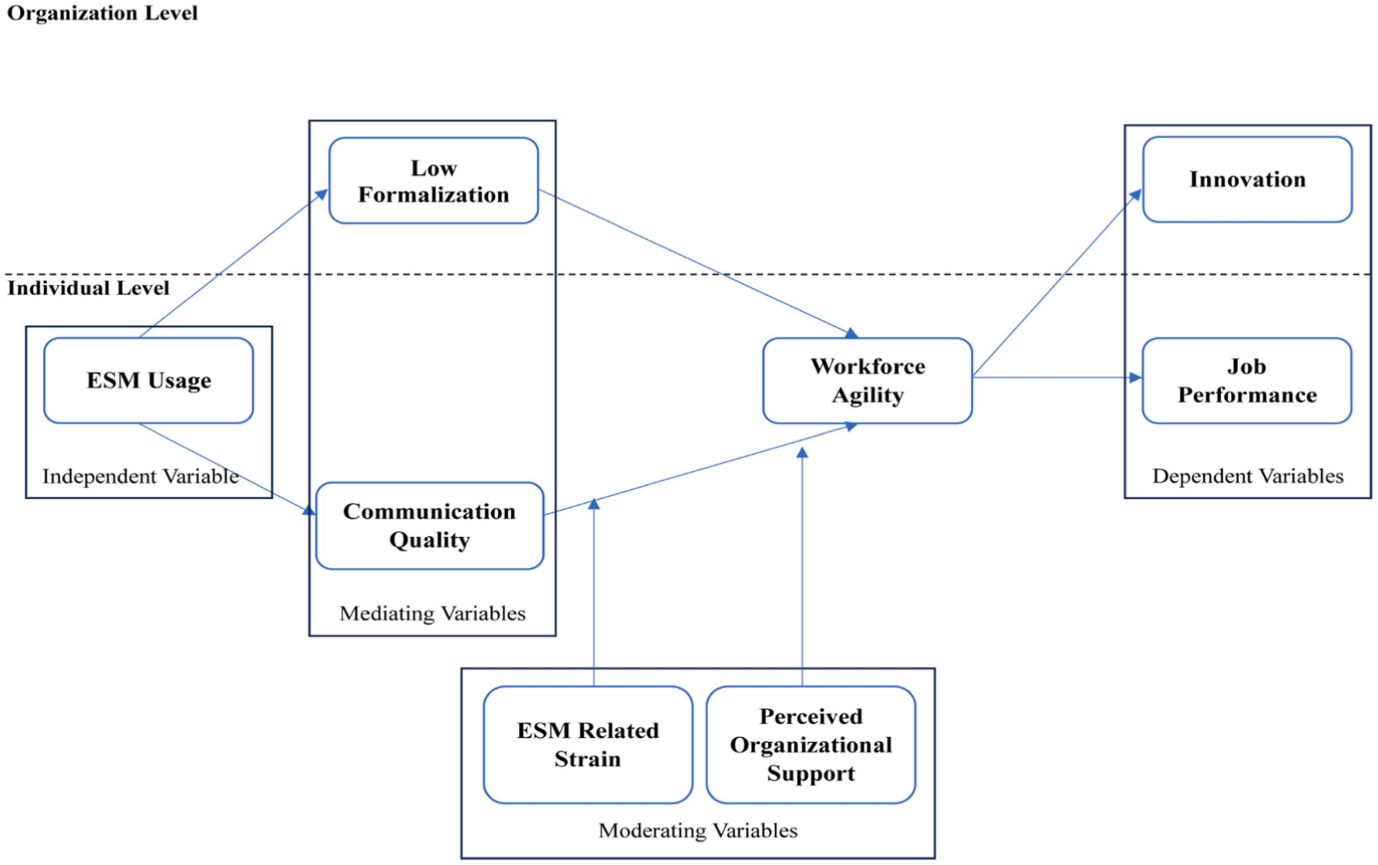
The visualization of the theoretical framework among variables related to workforce agility. Source: Data processed. ESM, enterprise social media.

We have developed a research framework by referring to previous research findings and integrating theories of management and organization and theories of communication and social interaction. The research framework for workforce agility has yet to explore multilevel analysis and moderation-mediation approaches. Therefore, we aim to offer a more comprehensive argument to advance future research (Figure 3).

The enterprise social media (ESM) usage (as an independent variable), which has been investigated six times, can influence communication quality and formalization levels (as mediating variables). The ESM usage tends to enhance information accessibility and facilitate more open and horizontal communication among individuals. It may also reduce formalization levels within the organizational hierarchy by promoting more informal relationships. High-quality communication can result in better understanding and more efficient coordination among employees. It potentially enhances workforce agility, which is the ability to adapt quickly to environmental changes. On the other hand, low formalization within the organization can also increase agility by allowing greater flexibility in responding to changes. However, the presence of moderating factors such as strain related to the use of corporate social media or perceptions of organizational support can influence the relationship between communication quality and workforce agility, although their role thus far has shown insignificant results.

First, ESM-related strain refers to the pressure or stress from ESM usage. In this context, high communication quality may impact workforce agility differently, depending on the extent of strain employees perceive regarding social media use. Suppose employees feel burdened or stressed by the amount of information to be processed or by the demands always to be connected and engaged in corporate social media. In that case, the influence of communication quality on workforce agility may be reduced or even reversed. Conversely, if the ESM-related strain is low, high communication quality may more strongly encourage employees to become more agile in responding to changes and effectively utilizing information. Second, perceived organizational support refers to employees’ perceptions of how much the organization supports and cares about their needs and well-being. In this context, perceived organizational support can moderate the relationship between communication quality and workforce agility. If employees feel supported by the organization, high communication quality may enhance their agility by strengthening understanding, trust, and coordination among team members and departments. Conversely, suppose employees feel less supported by the organization. In that case, the influence of communication quality on workforce agility may be less significant or even negative, as a lack of organizational support can inhibit employees’ ability to respond to changes and create innovative solutions quickly.

Overall, the resulting workforce agility is crucial in improving individual performance and innovation at the organizational level because it enables rapid adaptation to challenges and creates a dynamic work environment for idea exchange and collaboration.

### 4.3 Potential research gaps for future research avenues

The development of theories highlighting workforce agility reflects a significant evolution in our understanding of organizational dynamics and individual adaptation in rapidly changing and complex environments. To deepen our understanding of future workforce agility, researchers can explore the integration of existing theories with a more comprehensive and complex interdisciplinary approach. First, within the context of Organizational and Management Theory, researchers should explore how organizational support, work-based learning, and organizational memory formation influence workforce agility and how the complex interaction among these factors affects long-term company growth, innovation, and sustainability. Future research can explore the practical implications of these theories in the context of management practices and organizational strategic decision-making. Second, within the framework of Communication and Social Interaction Theory, the focus should be expanded to achieve a more comprehensive understanding of how open communication, formation and strengthening of strong social networks, as well as optimization of technology utilization, can enhance not only individual and organizational agility but also promote cross-border collaboration, innovation, and effective knowledge dissemination. Future research can explore more complex and adaptive communication designs and sophisticated technology utilization strategies to support adaptation, collaboration, and innovation in increasingly complex and interconnected workplaces. Third, Behavior and Learning Theory provides a rich foundation for understanding how individual adaptation to environmental and technological changes influences workforce agility and overall emotional and psychological well-being. Future research can deepen understanding of the complex and dynamic psychosocial factors that affect individual adaptation and identify and develop holistic and sustainable strategies to enhance emotional and psychological well-being in the workplace as a basis for sustainable agility. Lastly, within the framework of Economic Theory, research should delve deeper into the complex relationship between workforce agility and responses to changes in the global market, industry dynamics, and increasingly complex consumer demands. Future research can deepen understanding of how economic factors, including dynamic pricing strategies, investment policies, and global supply chain integration, influence business strategies, growth, and organizational sustainability in achieving optimal and sustainable agility in the face of increasing external uncertainty.

Previous research provided recommendations that can be used to identify gaps and provide directions for future research areas. The identification of recommendations in Table 5 is outlined as follows:

#### 4.3.1 Conducting longitudinal research

The application of longitudinal methods in researching workforce agility is highly recommended (Saleem et al., 2021; Zhu et al., 2021; Das et al., 2022; Zhang et al., 2022; Rasheed et al., 2023; Sun et al., 2023; Pitafi, 2024). This method facilitates observing changes and evolutions in behaviors and factors influencing agility over time. By incorporating a temporal dimension, this research can uncover trends, patterns, and changes in agility to provide more relevant insights.

Ajgaonkar et al. (2022) added to these recommendations by suggesting longitudinal quantitative comparative research across organizations and contextual settings. This method can offer valuable comparisons between organizations in different contexts, identifying differences and similarities in factors influencing agility. It can also provide a deeper understanding of how agility evolves and is relative to various organizational contexts.

#### 4.3.2 Integrating mixed methods

Integrating mixed methods in research design is a proposed method to gain a more holistic and detailed understanding of workforce agility phenomena. This mixed methods method leverages the strengths of quantitative analysis, providing insights into the extent to which specific factors contribute to the level of agility. Also, the qualitative method can capture nuances and specific contexts that may influence the interpretation and individual experiences of work agility (Park and Park, 2021; Hanu et al., 2023). By combining these methods, quantitative data can be used to identify general trends and patterns. At the same time, qualitative analysis can provide deeper insights through interviews, observations, or content analysis regarding the meaning and subjective experiences of employees related to agility.

#### 4.3.3 Considering the global-culture context

Expanding the research into a global context is a crucial step toward achieving a more comprehensive and culturally relevant understanding of workforce agility (Alavi and Wahab, 2017; Muduli and Pandya, 2018; Jannah, 2021; Lai et al., 2021; Soliman et al., 2021; Durst et al., 2023). It can be done by investigating workforce agility across various countries and industry sectors to explore contextual differences. Additionally, integrating diverse sectors such as public, manufacturing, banking, education, and SMEs in developing and developed countries is essential (Alavi and Wahab, 2017; Soliman et al., 2021).

A global method enables research to convey more contextual findings and understanding of the impact of cultural dynamics, regulations, and the economy on workforce agility practices. Meanwhile, the expansion of geographic and sectoral coverage provides a more comprehensive view of the factors influencing workforce agility at a global level. The results are expected to offer more valuable guidance for companies and organizations operating worldwide to develop effective agility strategies.

#### 4.3.4 Expanding samples

Incorporating various groups of respondents and introducing variation within the organizational context are significant steps to strengthen the research representation and enhance the generalizability of results. Saeed et al. (2022) suggested expanding the sample and geographic coverage, employing different research methods, and exploring mediating factors, as well as ensuring balanced participation of both males and females to gain a deeper understanding of spirituality in the workplace and employee agility in dynamic work environments. Goswami and Kumar (2018) proposed broadening the scope by examining the relationships between attributes and other dimensions for a more in-depth understanding of the factors influencing the overall performance of SMEs. Zandi et al. (2022) recommended including the telecommunications sector, banking sector, and developed countries to broaden the scope. Sameer (2022) suggested considering a longitudinal research design, exploring sectors other than the public sector, addressing low response rates, and investigating the impact of workforce agility related to individual factors, job design, as well as interactions in the context of blended working to provide in-depth insights into the continually evolving work environment. Sumukadas and Sawhney (2004) suggested rigorously testing the theory with a large and diverse sample, considering other contextual factors, and developing a measurement scale for workforce agility to understand the impact of team-based production incentives. Menon and Suresh (2022) recommended testing a conceptual model of workforce agility in various higher education institutions to expand triggers and attributes while broadening the sample. Al Hammouri et al. (2023) proposed investigating the impact of agile HRMS in the government sector, particularly in talent acquisition, learning and development, and employee engagement, to understand its influence on organizational performance and provide valuable insights for government agencies in optimizing their performance and effectiveness. Pitafi and Ren (2021) suggested focusing on the impact of ESM platforms on individual performance with a large sample, considering the work context and country, and including the workforce with minimal education. Oyen et al. (2001) recommended examining the potential for workforce agility in diverse production environments. By adopting these approaches, prior studies provided comprehensive and in-depth insights into their respective fields (Oyen et al., 2001; Sumukadas and Sawhney, 2004; Goswami and Kumar, 2018; Kumar Jha and Varkkey, 2018; Pitafi and Ren, 2021; Menon and Suresh, 2022; Saeed et al., 2022; Sameer, 2022; Zandi et al., 2022; Al Hammouri et al., 2023).

#### 4.3.5 Development conceptual model

Developing a conceptual framework in workforce agility research and adopting theoretical methods in designing the conceptual research framework have been repeatedly recommended by previous researchers (Breu et al., 2002; Herlina et al., 2021; Das et al., 2022; Maran et al., 2022; Salmen and Festing, 2022; Janani and Vijayalakshmi, 2023; Vuckovic et al., 2023; Muduli and Choudhury, 2024). A robust theory will better illustrate the relationships between variables and provide a solid theoretical foundation for interpreting results. Prior studies also showed the importance of further research on negative variables in the context of workforce agility. Factors such as conflict, stress, or burnout can play a significant role in understanding the complexity of the relationships between variables (Jin et al., 2020; Pitafi and Ren, 2021).

Workforce agility, which often involves the ability to quickly adapt to different changes and demands, can lead to significant role stress. Employees may feel pressured to fulfill various roles in a short amount of time, resulting in confusion about their responsibilities and role conflict. This, in turn, can lead to decreased job satisfaction and increased turnover. Additionally, the reliance on technology that often accompanies workforce agility can trigger technology stress. Employees may feel overwhelmed by the demands to constantly master new technologies and adapt to evolving systems. This technology stress can not only reduce productivity but also cause mental and emotional exhaustion. Fatigue is another significant negative impact of workforce agility. Employees who must continuously adapt to rapid changes and high demands tend to experience physical and emotional exhaustion. This fatigue not only affects individual performance but can also have detrimental effects on their mental and physical health. If not properly managed, fatigue can lead to burnout, which has the potential to reduce employee engagement and increase absenteeism.

Understanding the impact of agility on the negative variables can provide valuable insights for organizations, facilitating the development of balanced policies to enhance employee well-being and organizational effectiveness (Srivastava and Gupta, 2022). For instance, organizations can design more comprehensive training programs to reduce technology stress or implement flexible work policies to alleviate employee fatigue. Therefore, while workforce agility has many benefits, it is crucial for organizations to also address and manage the potential negative impacts. This will help create a healthier and more productive work environment, ultimately improving overall employee well-being.

#### 4.3.6 Exploring mediating and moderating variables

Exploring mediating and moderating variables is crucial for better understanding the relationships between variables. This method can provide insights into the mechanisms and contexts in which workforce agility develops (Talwar et al., 2023). Talwar et al. (2023) proposed considering affordability, additional impact and expanding the literature by exploring intervention mechanisms, such as mediation variables (organizational and relational factors) and moderation variables (network centrality, network size, frequency of ESM use, active vs. passive usage), to understand the transmission of the impact of ESM capabilities on agility. Heidt et al. (2023) suggested enhancing self-efficacy and locus of control in employee Human Resource Management (HRM) development to support the success of remote work, with organizational support as a key mediator. They also proposed conducting a multi-mediator analysis to understand the complex relationship between flexible work arrangements and future remote work success. The concept of multi-mediation refers to situations where the mediation of a relationship includes more than one mediator and the mediating effects of each mediator interact, reinforcing one another. Therefore, a multi-mediator analysis is necessary to understand the complex relationships of variables as mediators, which may result in a combined effect more significant than the individual effects. Lastly, Pitafi et al. (2019) recommended testing different mediating variables and extending the research to the team level.

#### 4.3.7 Development of workforce agility theory

Tamtam and Tourabi (2020) suggested the importance of understanding Breu et al. (2002) theoretical model regarding workforce agility and developing a more comprehensive assessment questionnaire. Additionally, they recommended conducting cross-organizational case studies and delving deeper into the factors influencing workforce agility. Sherehiy et al. (2007) proposed expanding the metrics and framework of agility in research, including literature on employee agility. They argued that a theoretical foundation was needed to identify the implications of agile management on workforce characteristics by establishing concepts and indicators of agility. Thayyib and Khan (2021) suggested considering more detailed measurements of workforce agility. Also, developing a more robust research instrument can enhance the validity and reliability of results, providing a more solid foundation for drawing conclusions and implications.

#### 4.3.8 The efficient use of evaluation methods

Franco and Landini (2022) identified data limitations and recommended future research with more robust designs, while Tessarini Junior and Saltorato (2021) suggested expanding data sources by searching other databases and considering subjective aspects in data source selection. Lim et al. (2021) proposed incorporating the restructuring of demographic profiles, utilizing the Partial Least Squares-Multi Group Analysis (PLS-MGA) method for group differences assessment, exploring comparisons with different populations, and applying similar models in other government departments. Muduli (2016) recommended expanding the sample and variations and validating through interviews. Qin and Nembhard (2010) advised considering additional sources of uncertainty, such as technological risks, and integrating quantitative and qualitative methodologies to understand workforce agility. Hopp and Van Oyen (2004) suggested developing a microeconomic framework to conceptualize further and advance this idea. In addition, Sameer (2022) and Wei et al. (2020) recommended using more efficient data collection methods, specifically from a managerial perspective. Research can provide management with quicker and more accurate information, facilitating informed decisions. Therefore, incorporating stakeholders in workforce agility research is crucial (Karl et al., 2021; Vuckovic et al., 2023). By understanding the perspectives and experiences of stakeholders, future research can more accurately reflect the needs and challenges of organizations in managing workforce agility, enhancing the acceptance and implementation of results in organizational practices.

#### 4.3.9 The implementation of a multilevel method

For exploring the dynamics of workforce agility, research may consider a multilevel method to comprehensively understand how factors at the individual and group/organizational levels interact (Pitafi et al., 2018, 2019; Wei et al., 2020; Jannah, 2021; Lim et al., 2021; Park and Park, 2021; Raut et al., 2022; Naim et al., 2023). It will provide deeper insights into the variability of agility among individuals and how organizational context influences employee behavior and attitudes. A multilevel method can help explore variability in workforce agility at both the individual and group levels.

Multilevel analysis, particularly concerning leadership as a group variable, provides new insights into crisis management (Naim et al., 2023). Research at the team level and testing other mediating variables are also necessary to better understand crisis response (Pitafi et al., 2019). Job characteristics, such as demands and supervisor support, are also considered in building employee agility, especially in social media usage at the team level (Pitafi et al., 2018). Cognitive flexibility at the individual and group levels is key to adapting to environmental changes (Park and Park, 2021). Further research at the organizational level is needed to understand overall organizational agility (Jannah, 2021). By considering demographic restructuring and using appropriate analytical methods, future research can be more beneficial in understanding agility in various contexts (Lim et al., 2021). Lastly, evaluating employee agility from a managerial perspective and studying team-level agility are crucial for a better understanding of the concept (Wei et al., 2020).

## 5 Limitations and future research directions

Although through a rigorous process of search and strict inclusion and exclusion criteria, we may inadvertently exclude some valuable publications from other databases, omitting unpublished books, conference proceedings, and dissertations, which could impact the results by overlooking additional articles containing relevant information on underlying concepts, relationships, and theories. Hence, future research can replicate this review by applying different search terms/criteria since we know each set of terms/criteria may cause selection biases (A’yuninnisa et al., 2023). Similarly, a high acceptance rate is suggested for hypotheses in literature (Heidt et al., 2023; Rasheed et al., 2023), leaving ambiguity about the truly ineffective aspects of workforce agility. It can be attributed to publication bias favoring articles with significant results. Therefore, scholars should openly address non-significant results in workforce agility research to offer valuable guidance for theory development and future investigations. It is also essential to explore contributions in languages other than English, providing a broader perspective but introducing the risk of including research with limited international impact. Moreover, empirical testing of the additional opportunities identified in this research within a theoretical framework can be a direction for future investigations.

## 6 Conclusion

In conclusion, an SLR approach was conducted in this study to consolidate knowledge of workforce agility, aiming to provide a unified concept of workforce agility and a comprehensive overview of related variables along with guiding theories. The review showed significant progress in workforce agility concerning concepts and research models (Pitafi et al., 2020; Lim et al., 2021; Zandi et al., 2022). This progress provides optimism for future investigations. However, a more structured method (such as conducting longitudinal research, adopting mixed methods, considering global-culture contexts, expanding samples, developing conceptual model, exploring mediation and moderation variables, developing workforce agility theory, and using more efficient methods and multilevel methods) is crucial for the development of this field.

## Data Availability

The original contributions presented in the study are included in the article/supplementary material, further inquiries can be directed to the corresponding author/s.
